# Supplementary material for: Digital wearable insole-based identification of knee arthropathies and gait signatures using machine learning
Source: eLife. 2024 Apr 30;13:e86132. doi: 10.7554/eLife.86132 (PMC11152572; doi:10.7554/eLife.86132)
Supplement: Supplementary file 1. [file elife-86132-supp1.docx]

Supplementary File 1 | Table of derived gait characteristics significant in differentiating control (HC) from knee osteoarthritis (OA) subjects. Columns present the characteristic, associated foot, average values in HC and OA, Wilcoxon test P and q values, SVM feature importance, category, and description. COP and GRF refer to center of pressure and ground reaction force, respectively.

| **Derived gait characteristic** | **Foot** | **Average value in HC** | **Average value in OA** | **OA v HC**   **wicoxon-test**   **nominal *P* value** | **OA v HC**   **wicoxon-test**   ***q***  **value** | **XGBoost feature importance** | **Category** | **Description** |
| --- | --- | --- | --- | --- | --- | --- | --- | --- |
| Bounding box of COP (AP/ML) (L) (Width) (mm) | left | 30.18 | 41.84 | 1.45E-10 | 5.39E-10 | 0.03 | COP |  |
| Force raise (L) (s) | left | 0.28 | 0.51 | 1.41E-17 | 1.29E-16 | 0.02 | dynamics | Time (in s) to first peak of the mean force curve after initial contact. Mean of all complete steps. |
| Force raise (R) (s) | right | 0.33 | 0.49 | 5.98E-10 | 1.96E-09 | 0.01 | dynamics |  |
| Forefoot/backfoot dominance (L) | left | 0.47 | 0.49 | 2.55E-08 | 5.65E-08 | 0.00 | coordination |  |
| max force (L) (N) | left | 13.97 | 11.93 | 1.03E-07 | 2.11E-07 | 0.10 | GRF |  |
| Mean COP (AP/ML) (R)   (y-direction) (mm) | right | -4.21 | -3.82 | 0.078 | 0.09 | 0.03 | COP |  |
| Mean COP velocity (R) (mm/s) | right | 396.67 | 312.29 | 2.08E-11 | 8.53E-11 | 0.09 | COP | Mean velocity of the center of pressure (COP) during stance phase. The faster the foot rolls off, the higher the gait line velocity. |
| Mean endpoint y of gait line (R) (mm) | right | -2.12 | 0.44 | 0.005 | 0.007 | 0.00 | gait line |  |
| Mean max force (R) (N) | right | 13.88 | 13.08 | 0.004 | 0.005 | 9.93E-05 | GRF | Maximum ground reaction force during stance phases. Mean of all complete steps |
| Mean start point x of gait line (L) (mm) | left | -93.25 | -76.25 | 1.19E-11 | 5.23E-11 | 0.02 | gait line |  |
| Mean start point x of gait line (R) (mm) | right | -96.78 | -82.78 | 4.28E-09 | 1.06E-08 | 0.00 | gait line |  |
| Medial-/lateral Dominance (L) | left | 0.47 | 0.46 | 0.4572 | 0.4746 | 0.01 | coordination |  |
| Medial-/lateral Dominance (R) | right | 0.47 | 0.49 | 0.0001 | 0.0002 | 0.00 | coordination |  |
| sd of stance duration (R) (s) | right | 0.07 | 0.10 | 5.18E-08 | 1.12E-07 | 0.00 | temporal | When standard deviations are given, then the notion is always "SD across steps". These values describe the variability of gait. |
| sd of swing duration (R) (s) | right | 0.03 | 0.06 | 3.06E-10 | 1.09E-09 | 0.02 | temporal |  |
| sd x of gait line start point (L) (mm) | left | 3.98 | 12.95 | 1.35E-19 | 2.21E-18 | 0.09 | gait line |  |
| sd x of gait line start point (R) (mm) | right | 3.26 | 13.04 | 5.21E-20 | 1.07E-18 | 0.06 | gait line |  |
| sd y of gait line start point (L) (mm) | left | 0.87 | 2.07 | 1.07E-18 | 8.94E-17 | 0.02 | gait line |  |
| sd y of gait line start point (R) (mm) | right | 0.86 | 2.24 | 4.61E-18 | 6.31E-17 | 0.00 | gait line |  |
| Take-off dynamics (L) | left | 1.16 | 0.26 | 1.52E-13 | 1.04E-12 | 0.28 | dynamics | Time to first peak of the mean force curve after initial contact. Mean of all complete steps. |
| Take-off dynamics (R) | right | 1.14 | 0.36 | 4.10E-09 | 1.05E-08 | 0.20 | dynamics |  |

COP, center of pressure; GRF, ground reaction force; HC, controls.
